# Strategy to develop a MAO-A-resistant 5-hydroxy-l-[β-^11^C]tryptophan isotopologue based on deuterium kinetic isotope effects

**DOI:** 10.1186/s13550-014-0062-2

**Published:** 2014-11-30

**Authors:** Jonas Eriksson, Ola Åberg, Ram Kumar Selvaraju, Gunnar Antoni, Lars Johansson, Olof Eriksson

**Affiliations:** Preclinical PET Platform (PPP), Department of Medicinal Chemistry, Uppsala University, Dag Hammarskjölds väg 14C, 3tr, SE-751 83 Uppsala, Sweden; PET Centre, Uppsala University Hospital, SE-751 85 Uppsala, Sweden; Department of Radiology, Oncology and Radiation Sciences, Uppsala University, Uppsala University Hospital, SE-751 85 Uppsala, Sweden; AstraZeneca R&D, Pepparedsleden 1, SE-431 50 Mölndal, Sweden

**Keywords:** 5-Hydroxy-tryptophan, [^11^C]HTP, Kinetic isotope effect, Deuterium, Neuroendocrine tumors, Beta cell imaging

## Abstract

**Background:**

The serotonin precursor 5-hydroxy-l-[β-^11^C]tryptophan ([^11^C]HTP) is in clinical use for localization of neuroendocrine tumors and has been suggested as a proxy marker for pancreatic islet cells. However, degradation by monoamine oxidase-A (MAO-A) reduces retention and the contrast to non-endocrine tissue.

**Methods:**

A synthesis method was developed for 5-hydroxy-l-[β*-*^11^C^2^H]tryptophan ([^11^C]DHTP), an isotopologue of [^11^C]HTP, labeled with ^11^C and ^2^H at the β-position adjacent to the carbon involved in MAO-A decarboxylation. MAO-A-mediated degradation of [^11^C]DHTP was evaluated and compared to non-deuterated [^11^C]HTP.

**Results:**

[^11^C]DHTP was synthesized with a radiochemical purity of >98%, radioactivity of 620 ± 190 MBq, and deuterium (^2^H or ^2^H_2_) incorporation at the β-position of 22% ±5%. Retention and resistance to MAO-A-mediated degradation of [^11^C]DHTP were increased in cells but not in non-human primate pancreas.

**Conclusions:**

Partial deuteration of the β-position yields improved resistance to MAO-A-mediated degradation *in vitro* but not *in vivo*.

## Background

Non-invasive assessment of the serotonergic biosynthesis by 5-hydroxy-l-[β-^11^C]tryptophan ([^11^C]HTP) is an established PET marker for many forms of neuroendocrine tumors (NETs) [1] and has recently been suggested as a surrogate marker for the neuroendocrine pancreas [2-4].

Di Gialleonardo et al. showed that [^11^C]HTP accumulation in a pancreatic neuroendocrine cell line was significantly increased by blocking the major route of degradation, monoamine oxidase-A (MAO-A) [2]. Furthermore, this mode of degradation was not present in a ductal cell line. The authors therefore posit that an HTP analogue, following the formation of 5-hydroxy-[^11^C]tryptamine ([^11^C]5-HT) by aromatic amino acid decarboxylase (AADC), resistant to MAO-A-mediated degradation will have an increased retention in neuroendocrine tissues containing the amine precursor uptake and decarboxylation (APUD) mechanism. APUD cells include not only the islets of Langerhans in the pancreas but in a broader scope also many forms of NETs. In fact, [^11^C]HTP-PET was originally developed for assessment of the AADC step in serotonin biosynthesis but has found extensive use as a highly sensitive diagnostic tool for primary or metastatic NETs [1]. However, the contrast to surrounding tissues and hence the sensitivity are affected by the rapid MAO-A-mediated conversion of [^11^C]5-HT into 5-hydroxy-[^11^C]indoleacetic acid ([^11^C]5-HIAA). Current strategies to improve contrast involve peroral administration of the AADC inhibitor carbidopa, which increases the biological half-life of the tracer by reducing peripheral metabolism, e.g., in the liver. However, this method induces large individual variations in tracer metabolism and precludes comparison between examinations and therefore therapy efficacy monitoring [5]. Increased retention of [^11^C]HTP in NETs could improve the sensitivity of examination in addition to potentially visualizing smaller NETs currently below detection threshold.

Development of new HTP analogues with improved resistance to MAO-A degradation and with retained affinity to AADC is a difficult challenge [6]. A straightforward approach to potentially alter metabolic stability while retaining [^11^C]HTP structurally congruent to biogenic HTP is to isotopically substitute the compound with deuterium in a position that would induce a kinetic isotope effect (KIE) large enough to deter MAO-A interaction. Similar approaches have previously been employed to improve pharmacodynamics properties of radiotracers and to increase metabolic stability against unwanted degradation which impairs visualization of a target tissue [7-9].

Deuterium substitution at the β-carbon adjacent to the reaction center involved in the deamination pathway would typically be expected to give a minor KIE of 1.1 to 1.2. While this effect would not be expected to be particularly strong, the β-position was predicted to be readily accessible for ^11^C- and ^2^H-labeling by only minor changes to the original synthesis of [^11^C]HTP, and thus, it would serve as a convenient test case to explore the means of deuteration to improve MAO-A resistance. A more potent position could be deuteration at the α-carbon, which is expected to yield a primary isotope effect that is significantly higher than the secondary KIE. However, this would require that the deuterium label in α-position remain intact also after conversion by AADC to 5-HT, which is uncertain. [1-*N*-^11^C, α,α-^2^H_2_]l-deprenyl, a selective MAO-B inhibitor, has previously been reported to induce a KIE of 3.8 ± 1.1 in baboon brain [9].

Here, we present the radiosynthesis of 5-hydroxy-l-[β-^11^C^2^H]tryptophan ([^11^C]DHTP) and the preclinical evaluation of its stability in regard to MAO-A *in vitro* and *in vivo*, in a direct comparison with [^11^C]HTP.

## Methods

### Radiochemistry

[^11^C]HTP was synthesized according to the method by Bjurling et al. [10]. The method was modified to produce [^11^C]DHTP by exchanging the ^11^C-labeled reagent [^11^C]methyl iodide for [^11^C^2^H_3_]methyl iodide. The deuterated [^11^C]methyl iodide was obtained by reduction of [^11^C]carbon dioxide with LiAl^2^H_4_ followed by treatment with hydriodic acid at standard reaction conditions used for [^11^C]methyl iodide production [11].

Purification was performed by semi-preparative high-performance liquid chromatography (VWR LaPrep HPLC, VWR, Radnor, PA, USA) using an Ultrasphere ODS column (Beckman, Brea, CA, USA, 5 μm, 250 × 10 mm) with eluents A: acetic acid (17 mM)/ascorbic acid (1 mM), and B: ethanol (99.5%). After purification, the eluents were removed using rotary evaporation and the product was reformulated in 10 mL pharmaceutical-grade solution containing ethanol 99.5%/phosphate buffer/water (0.9/2.3/6.8) and sterile filtered (Dynagard, 0.2 μm, 2.5 cm^2^ Hollow Fiber Syringe Filter, Microgon Inc., Laguna Hills, CA, USA). A sample was analyzed by analytical HPLC (VWR LaChrom Elite) using a Spherisorb column (Waters, Milford, MA, USA, 5 μm, 4.6 × 250 mm) and eluents A: acetic acid (17 mM), and B: acetonitrile. Both HPLC systems were equipped with UV detectors set at 254 nm and radio detectors (Bioscan Flow-Count PMT, Bioscan, Washington, DC, USA).

Deuterium incorporation in [^11^C]DHTP was determined using liquid chromatography-electrospray ionization-mass spectrometry (LC-ESI-MS; Micromass Quattro Premier triple quadrupole mass spectrometer, Waters) with positive mode scanning and selected ion recording *m*/*z* 221.1 (=HTP + H), 222.1 (=[^2^H]HTP + H), and *m*/*z* 223.1 (=[^2^H_2_]HTP + H). Data was corrected for the natural abundance of ^13^C.

### Cell studies

INS-1 cells (an insulinoma cell line derived from rat, 0.185 × 10^6^ cells) or PANC1 cells (a non-endocrine line derived from human ductal cells, 0.1 × 10^6^ cells) were incubated with 1 to 2 MBq [^11^C]HTP or [^11^C]DHTP in 1 mL PBS for 15, 30, or 60 min at 37°C either alone (baseline) or together with 10 μM carbidopa (inhibitor of AADC, Karbidopa, Apoteket AB, Stockholm, Sweden), 10 μM HTP, or 10 μM clorgyline (inhibitor of MAO-A, Sigma, St. Louis, MO, USA). All samples were repeated in triplicate.

After incubation, the cells were filtered using washing buffer Tris-HCl (50 mM, pH 7.4) through a Whatman GF/C filter paper (pore size 1.2 μm; Semat International Ltd., St. Albans, UK), using a cell harvester (Brandel, Gaithersburg, MD, USA). The radioactivity of the cells trapped on the filter was measured in a NaI (Tl) well counter (Uppsala Imanet AB, Uppsala, Sweden), and the cellular radioactive uptake was corrected for radioactive decay. Radioactive uptake was expressed as percent of incubation dose (%ID) per million cells.

### *Ex vivo* organ distribution in rats

Sprague Dawley rats (*n* = 26, weighing 392 ± 68 g) were housed under standard laboratory conditions with free access to food and water. All handling and experiments were carried out in accordance with the national guidelines and were approved by the local ethics committee for animal research. All applicable institutional and/or national guidelines for the care and use of animals were followed.

Rats under general isoflurane anesthesia (3.6%) were administered with 12.0 ± 2.1 MBq [^11^C]HTP (*n* = 16) or 9.4 ± 0.6 MBq [^11^C]DHTP (*n* = 11) intravenously into the tail vein. Subgroups of groups receiving each tracer (*n* = 5 for [^11^C]DHTP and *n* = 7 for [^11^C]HTP) were pre-administered with 2 mg/kg clorgyline 10 min prior to tracer injection to inhibit MAO-A-mediated degradation.

The animals were allowed to wake up following tracer administration and were euthanized by CO_2_ 30 or 60 min following injection. Tissues of interest were immediately excised, and the radioactivity concentrations were measured in a NaI (Tl) well counter and related to the administered amount of tracer and the animal weight by standardized uptake values (SUV) to allow for comparison between animals.

### Cynomolgus PET/CT

The non-human primate experiments were carried out in accordance with the national guidelines and were approved by the local ethics committee for animal research. All applicable institutional and/or national guidelines for the care and use of animals were followed. Cynomolgus monkeys (*n* = 2, 7,000 and 8,900 g) were examined in this study. Anesthesia was performed as described previously [3]. The results of the [^11^C]HTP examination have been published previously [3], but its inclusion here is merited as a direct within-individual comparison with [^11^C]DHTP.

The target organ in the studies was the neuroendocrine pancreas, due to its importance in diabetes. For all examinations, 8.6 to 15.7 MBq/kg [^11^C]HTP or [^11^C]DHTP was administered intravenously and the animals were examined over the abdomen by a dynamic PET protocol for 90 min (33 frames; 12 × 10, 6 × 30, 5 × 120, 5 × 300, and 5 × 600 s). Each animal was examined by both [^11^C]HTP and [^11^C]DHTP under baseline conditions in the morning. After baseline examinations, each animal was administered intravenously with 2 mg/kg clorgyline (Sigma) dissolved in 0.9% NaCl to selectively and irreversibly inhibit MAO-A. Each animal was examined again with [^11^C]HTP and [^11^C]DHTP 0.5 or 2.5 h later. The reason for these time points is first to allow clorgyline to bind to MAO-A for 0.5 h. The first isotopologue examination (including dynamic abdominal scanning and a whole-body scan) then takes 2 h. After this time (six half-lives), almost no radioactivity from the first administration remains and the second isotopologue can be administered. To avoid bias in the change in effect of MAO-A inhibition over time, individual 1 was examined first with [^11^C]HTP and then [^11^C]DHTP, while individual 2 was examined in the opposite order.

Venous blood samples (0.2 mL) were collected at 0.5, 1, 3, 5, 10, 15, 20, 30, 45, 60, and 90 min after each injection to measure the radioactivity concentration in whole blood and plasma. Blood samples (1.5 to 2.5 mL) were drawn at 5, 30, and 60 min following tracer administration for analysis of tracer metabolites in plasma.

### Tracer metabolite analysis of [^11^C]DHTP and [^11^C]HTP

The blood sample was centrifuged at 4,000 rpm for 2 min at 4°C (Beckman Allegra X-22R Centrifuge, Beckman Coulter Inc., Palo Alto, CA, USA). One milliliter of plasma was taken and 1.0 mL of 7 *w*/*v* % perchloric acid was added to precipitate the proteins. The mixture was centrifuged at 13,200 rpm at 4°C for 1 min (Eppendorf 5415R centrifuge, Eppendorf AG, Hamburg, Germany). The supernatant was filtered through a 0.2-μm nylon membrane (Corning Incorporated, Corning, NY, USA) by centrifugation at 13,200 rpm at 4°C for 1 min. Ten microliters of unlabeled 1 mg/mL HTP, HIAA, and serotonin (5-HT) was added to the supernatant. The parent compound and metabolites were separated by HPLC and fractionated based on the UV signals from the added unlabeled compounds. The percentage intact tracer was then assessed by measuring the activity in each fraction using a NaI (Tl) well counter (Uppsala Imanet AB) and correcting for radioactive decay. The sample preparation recovery was determined by measuring the radioactivity in the plasma, filters, and the pellet.

### Image analysis

Image acquisition was performed in three-dimensional (3D) mode and reconstructed using an iterative OSEM VUEPOINT algorithm (2 iterations/21 subsets, in a 128 × 128 matrix, zoom 50 cm in diameter). Reconstructed data were analyzed by PMOD (PMOD Technologies Ltd., Zurich, Switzerland). Regions of interest (ROIs) were delineated on co-registered transaxial CT slices. Entire organs, when possible, were delineated on sequential slices and combined into VOIs. Organ uptake was expressed as SUV.

### Statistics

Results are expressed as averages ± standard deviation throughout. Comparison between groups was assessed by Student's *t*-test. For the cell experiments, the linear regression curves and subsequent comparisons in elevation and slope were performed in GraphPad Prism 5 (GraphPad, La Jolla, CA, USA). *P* < 0.05 was considered significant.

## Results

### Radiochemistry

[^11^C]HTP and [^11^C]DHTP were reformulated in sterile solutions ready for injection containing 740 ± 240 and 620 ± 190 MBq, respectively, with a radiochemical purity of more than 98%. The identities of the synthesized radiolabeled compounds were confirmed by comparison of retention times with authentic HTP using HPLC equipped with UV and radio detectors. The deuterium incorporation in [^11^C]DHTP was determined by LC-MS; 15.0% ±2.9% contained one ^2^H, and 7.1% ±1.8% contained two ^2^H. The total fraction ^2^H-substituted [^11^C]DHTP was 22.1% ±4.7%.

### Cell studies

**Figure 1:**
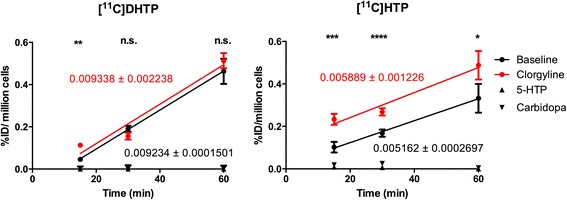
**Uptake and retention in beta cells of [** ^**11**^ **C]DHTP (left panel) and [** ^**11**^ **C]HTP (right panel).** [^11^C]DHTP had increased retention in the neuroendocrine cell line INS-1 compared to [^11^C]HTP as measured by the slope of linear regression. Inhibition of MAO-A increased the retention of [^11^C]HTP at all time points, but only at a single time point for [^11^C]DHTP. Stars indicate increase of tracer uptake after MAO-A inhibition as compared to baseline.

[^11^C]DHTP retained affinity for AADC as its uptake in INS-1 was completely abolished by co-incubation with either carbidopa or HTP (Figure 1, left panel) similarly to non-deuterated [^11^C]HTP. Inhibition of MAO-A had low effect on [^11^C]DHTP uptake or retention (no effect on elevation or slope of linear regression, and only significant increase at the 15 min time point *p* < 0.05) compared to baseline, while uptake of [^11^C]HTP was increased at all time points (each individual time point *p* < 0.05, as well as elevation of linear regression *p* < 0.05). The retention of [^11^C]DHTP was significantly increased compared to that of [^11^C]HTP (slope of linear regression *p* < 0.01). There was no difference in retention between [^11^C]DHTP at baseline conditions and [^11^C]HTP during MAO-A inhibition.

Negligible uptake (unaffected by inhibition of AADC or MAO-A or by competition by unlabeled HTP) was seen of either isotopologue in non-neuroendocrine PANC1 cells (data not shown).

### *Ex vivo* organ distribution in rats

**Figure 2:**
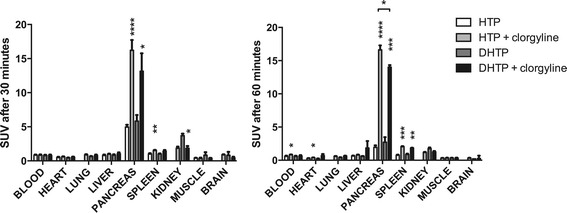
**Direct comparison of biodistribution of [** ^**11**^ **C]DHTP and [** ^**11**^ **C]HTP in Sprague Dawley rats following radiotracer administration.** Left panel: 30 min. Right panel: 60 min. Baseline uptake of [^11^C]DHTP in the endocrine pancreas had a tendency to increase compared to that of [^11^C]HTP. MAO inhibition had reduced effect on [^11^C]DHTP compared to [^11^C]HTP. Stars indicate level of significance (*p < 0.05; **p < 0.01; ***p < 0.001; ****p < 0.0001).

There was a moderate increase in pancreatic uptake (17% at 30 min and 40% at 60 min) of [^11^C]DHTP compared to [^11^C]HTP, but the increase was not statistically significant. As shown previously, there is a marked increase in retention of [^11^C]HTP in the neuroendocrine pancreas after inhibition of MAO-A (Figure 2, *p* < 0.0001 after 30 and 60 min). An increase in pancreatic retention of [^11^C]DHTP was also seen after MAO inhibition. However, the effect on [^11^C]DHTP was significantly lower than that on [^11^C]HTP after 60 min (16%, *p* < 0.05).

### Cynomolgus PET/CT

**Figure 3:**
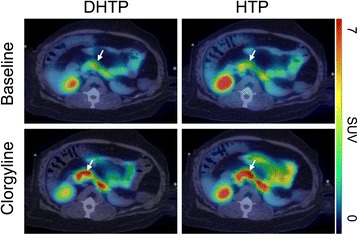
**Transaxial view of the abdomen of individual 2.** Following intravenous administration of [^11^C]DHTP (top left panel) or [^11^C]HTP (top right panel) during baseline conditions and following intravenous pre-treatment by 2 mg/kg clorgyline (bottom panels). Images are summations of examinations between 10 and 90 min and normalized to SUV = 7 (red in the scale bar to the right). The [^11^C]HTP examinations have been published previously at group level [3]. White arrows indicate the location of the pancreas.

**Figure 4:**
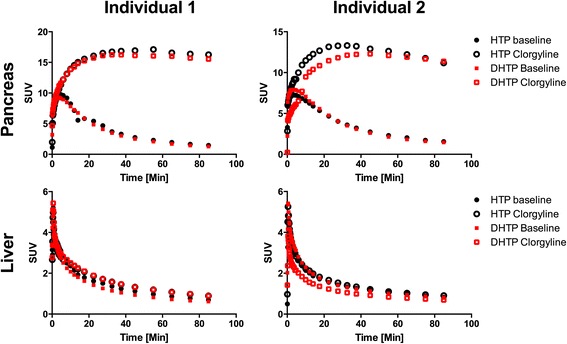
**Dynamic PET examinations of individual 1 (left panels) and individual 2 (right panels).** The examinations reveal rapid high accumulation in the pancreas of both [^11^C]DHTP and [^11^C]HTP followed by washout which is completed after approximately 60 min (top panels). Selective inhibition of MAO-A reduces washout of both analogues to a minimum. [^11^C]DHTP and [^11^C]HTP are selective for neuroendocrine tissues, and consequently, no accumulation (under baseline conditions) or effects from inhibiting MAO-A are seen in tissues such as the liver (bottom panels). The [^11^C]HTP examinations have been published previously at group level [3].

We have previously shown that pancreatic uptake of [^11^C]HTP in non-human primates is directly dependent on conversion into [^11^C]5-HT by AADC and that degradation and washout are mediated by MAO-A [1]. Here, the neuroendocrine pancreas was clearly visualized by both [^11^C]DHTP and [^11^C]HTP both during baseline conditions and following pre-treatment by clorgyline (Figure 3). The dynamic time-dependent uptake of the isotopologues was virtually identical in both individuals (Figure 4, top panels). Inhibition of MAO-A abolished the washout from the pancreas throughout the duration of the examinations, and there was no appreciable difference in the effect of MAO-A inhibition between the deuterated and the non-deuterated isotopologues. MAO-A inhibition had no effects on retention of either [^11^C]DHTP or [^11^C]HTP in non-neuroendocrine tissues such as the liver (Figure 4, bottom panels).

There was no statistical increase in the percentage of intact [^11^C]DHTP compared to [^11^C]HTP in blood plasma in either of the individuals (Table 1). MAO-A inhibition increased the percentage of intact radiotracer in plasma compared to baseline, but again there was no statistical difference between the isotopologues.

**Table 1:** **The percentage of intact parent compounds in blood plasma in non-human primates**

	**Individual 1**				**Individual 2**			
	**Baseline**		**MAO-A inhibited**		**Baseline**		**MAO-A inhibited**	
**Time (min)**	**[** ^**11**^ **C]HTP**	**[** ^**11**^ **C]DHTP**	**[** ^**11**^ **C]HTP**	**[** ^**11**^ **C]DHTP**	**[** ^**11**^ **C]HTP**	**[** ^**11**^ **C]DHTP**	**[** ^**11**^ **C]HTP**	**[** ^**11**^ **C]DHTP**
5	89.1	89.1	95.1	95.2	92.0	90.6	95.0	96.2
30	65.0	66.7	73.3	72.6	70.1	68.9	77.0	80.6
60	50.0	53.1	54.3	58.8	56.0	52.3	63.2	65.2

## Discussion

Improved resistance to MAO-A will potentially yield markedly increased intracellular retention of [^11^C]HTP in neuroendocrine tissues. [^11^C]HTP is currently used as a universal diagnostic imaging biomarker for benign and malignant NETs [1]. However, a higher amount of intact [^11^C]HTP in plasma has been shown to improve the tracer uptake in NETs, and patients therefore are pre-medicated with the AADC inhibitor 100 to 200 mg carbidopa orally 1 h prior to the PET examination [5]. This procedure not only improves sensitivity but also introduces a new parameter that directly influences absolute uptake in lesions: the individual time for gastrointestinal absorption of carbiodopa. Additionally, the time from ingestion of carbidopa until PET examination can be variable in case of delays in radiochemistry. This taken together makes direct quantitative comparison between examinations - even repeated scans within an individual - impossible or difficult. Importantly, it precludes the possibility of quantitatively monitoring treatment efficacy by using SUVs in individual lesions.

One could consider to induce an increase in retention of [^11^C]HTP in NETs as seen in the endocrine pancreas in Figures 3 and 4 by pre-treating patients with a clinically approved MAO-A inhibitor such as moclobemide. This would likely improve sensitivity substantially, but peroral administration would introduce similar individual variation to that in carbidopa. Intravenous administration on a routine basis is counter-indicated due to the risk of causing serotonergic syndrome due to elevated 5-HT levels in the CNS. Theoretically, development of non-brain-penetrant MAO-A inhibitors could potentially provide an alternative way forward, but we instead suggest minimal re-design of the biomarker itself to achieve the same goal in an elegant manner.

**Scheme 1:**
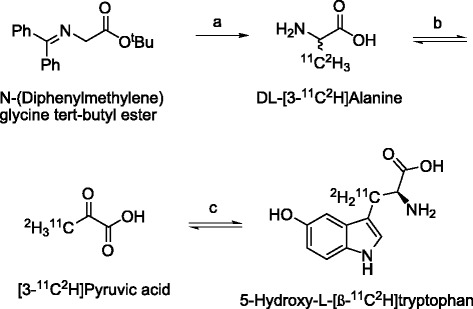
**Radiosynthesis of [** ^**11**^ **C]DHTP. (a)** *i* [^11^C_2_H_3_]methyl iodide, KOH, DMF; *ii* solvent removal by SPE; *iii* HCl. **(b)** Tris, pH 8.3 to 9.0, d-amino acid oxidase (D-AAO)/catalase, glutamic pyruvic transaminase (GTP). **(c)** *i* Tryptophanase (TPase), 5-hydroxyindole; *ii* HCl; *iii* filtration; *iv* semi-preparative HPLC purification.

The modification to the previously published [^11^C]HTP synthesis method to obtain [^11^C]DHTP was straightforward and involved only change in the [^11^C]methyl iodide synthesis where LiAlH_4_ was replaced with LiAl^2^H_4_. Despite possible KIEs throughout the different synthesis steps, there was no significant difference in radiochemical yields between [^11^C]HTP and [^11^C]DHTP (740 ± 240 vs. 620 ± 190 MBq, Scheme 1). LC-MS analysis showed that the incorporation yield of ^2^H was moderate for [^11^C]DHTP with 22% containing either one or two ^2^H. Generally, labeling using deuterated methyl iodide is expected to give higher incorporation of deuterium in the final product [12], but in this case, the reversible enzymatic reaction from pyruvate to hydroxytryptophan is suspected to induce loss of deuterium label. The degradative process that transforms 5-hydroxy-l-tryptophan back to pyruvate proceeds over α-aminoacrylate, the same enzyme-bound intermediate as in the synthetic process [13]. The reversibility allows addition of ^1^H at the deuterated β-position in pyruvate and consequently also loss of ^2^H when converted back again to hydroxytryptophan.

**Scheme 2:**
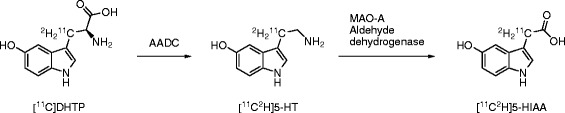
**Theoretical metabolism of [** ^**11**^ **C]DHTP in neuroendocrine cells.** [^11^C]DHTP is converted into [^11^C^2^H]5-HT by AADC. Deamination by MAO-A, and the subsequent cellular washout in the form of [^11^C^2^H]5-HIAA, is theoretically reduced by a secondary KIE.

Cell studies showed that [^11^C]DHTP had increased retention compared to [^11^C]HTP (Scheme 2). Furthermore, inhibition of MAO-A by clorgyline had less effect on [^11^C]DHTP as opposed to [^11^C]HTP. The effect still seen (for example, at the 15 min time point) can still be expected given that only a partial deuteration was achieved. Importantly, inhibition of MAO-A increased the retention of [^11^C]HTP to the levels seen for [^11^C]DHTP under baseline conditions. This suggests that [^11^C]DHTP has improved stability in regard to MAO-A-mediated degradation *in vitro*, while retaining the affinity for AADC which is crucial for preserving high sensitivity for imaging of neuroendocrine tissues.

After these promising results in cells, we investigated the biodistribution of [^11^C]DHTP in rodents and especially the effect of inhibition of MAO-A. Increased resistance to MAO-A should in theory result in increased retention in tissues, which facilitates serotonin to a high degree (i.e., the neuroendocrine pancreas in this study). We found a tendency for increased pancreatic uptake of the deuterated isotopologue, but this was not a significant change. [^11^C]DHTP retention in the pancreas was still markedly increased by inhibition of MAO-A, but to a lesser degree than [^11^C]HTP. These results show that the current formulation of [^11^C]DHTP is less sensitive to, but still to a considerable extent metabolized by, MAO-A in rodents. Given that only 25% of the [^11^C]DHTP was deuterated and still increased retention by 17% to 40%, we estimate that a formulation of 100% deuterated [^11^C]DHTP would improve the retention in rodent pancreas by approximately 70% to 160%. More substantial resistance against MAO-A, which may be reached by deuteration at the α-position, potentially increases the absolute uptake by up to threefold after 30 min and almost tenfold after 60 min (as seen when inducing complete inhibition of MAO-A by clorgyline, Figure 3).

In non-human primates, however, there was no measurable difference between [^11^C]DHTP and [^11^C]HTP regarding baseline accumulation and retention or following MAO-A inhibition. Here, as in the rodent, we can see the potential of reducing degradation against MAO-A, as retention of [^11^C]HTP in the endocrine pancreas is increased by tenfold by inhibition by MAO-A [3]. A similar increase in retention in APUD tissues will clearly yield a significant improvement in diagnostic sensitivity in the oncological setting.

PET imaging measurement data will invariably have lower sensitivity and more noise than data acquired by well counter measurements (in this case, *ex vivo* organ distribution studies and *in vitro* cell studies). This is conceivably one of the reasons for the lack of measurable effect in non-human primates despite promising *in vitro* and small-animal *in vivo* results.

As we did not see any noticeable increased endocrine retention of tracer in the PET study in non-human primates - most likely the most predictable for the clinical situation - we decided against further evaluation in a neuroendocrine xenograft mouse model since it conceivably could yield ‘false’ positive results in small-animal PET imaging and *ex vivo* organ distribution studies, which would not translate to imaging using less sensitive clinical PET scanners.

We conclude that the present formulation of [^11^C]DHTP (22.1% ±4.7% deuterated) is still to a major extent degraded by MAO-A in non-human primates and that the secondary KIE introduced by a partial deuteration of the β-position has only a minor effect on the stability of [^11^C]DHTP against this enzyme despite promising results in cells and in rodents. The small effect is likely due to the low proportion of deuterated molecules, and it follows that increasing the deuteration efficiency at the β-position, or direct labeling of the α-position, may yield improved MAO-A resistance.

## Conclusions

The current study shows the feasibility of improving the metabolic *in vivo* stability of [^11^C]HTP by minimal re-design. Partial deuteration of the β-position yields improved resistance to MAO-A-mediated degradation in INS-1 cells and in rodent pancreas *in vivo*, but no appreciable increased retention in non-human primate neuroendocrine tissue. In future studies, we aim to improve labeling efficiency with respect to deuteration of the β-position and attempt deuteration of the α-position to further improve the MAO-A resistance of [^11^C]HTP and increase its sensitivity as a neuroendocrine marker.

## Abbreviations

[^11^C]5-HIAA: 5-hydroxy-[^11^C]indoleacetic acid
[^11^C]5-HT: 5-hydroxy-[^11^C]tryptamine
[^11^C]DHTP: 5-hydroxy-l-[β-^11^C^2^H]tryptophan
[^11^C]HTP: 5-hydroxy-l-[β-^11^C]tryptophan
AADC: aromatic amino acid decarboxylase
APUD: amine precursor uptake and decarboxylation
KIE: kinetic isotope effect
MAO-A: monoamine oxidase-A
NET: neuroendocrine tumors
ROI: region of interest
